# Risk Factors for Working Pregnant Women and Potential Adverse Consequences of Exposure: A Systematic Review

**DOI:** 10.3389/ijph.2023.1605655

**Published:** 2023-02-16

**Authors:** María del Rocío Corchero-Falcón, Juan Gómez-Salgado, Juan Jesús García-Iglesias, Juan Carlos Camacho-Vega, Javier Fagundo-Rivera, Ana María Carrasco-González

**Affiliations:** ^1^ Faculty of Labour Sciences, University of Huelva, Huelva, Spain; ^2^ Department of Sociology, Social Work and Public Health, Faculty of Labour Sciences, University of Huelva, Huelva, Spain; ^3^ Safety and Health Postgraduate Programme, Universidad Espíritu Santo, Guayaquil, Ecuador; ^4^ Department of Building Construction II, Higher Technical School of Building Engineering, University of Seville, Seville, Spain; ^5^ Centro Universitario de Enfermería Cruz Roja, University of Seville, Seville, Spain; ^6^ Department of Social, Developmental and Educational Psychology, University of Huelva, Huelva, Spain

**Keywords:** pregnancy, risk factors, work environment, work stress, adverse birth outcomes

## Abstract

**Objective:** To assess the risk factors perceived as stressors by pregnant women in the work environment and the possible adverse consequences of such exposure for the normal development of pregnancy.

**Methods:** Systematic review, guided by the PRISMA guidelines, and using Pubmed, Web of Science, Dialnet, SciELO, and REDIB databases. Methodological quality was assessed using the critical appraisal tools for non-randomised studies of the Joanna Briggs Institute.

**Results:** A total of 38 studies were included. The main risk factors found in the work environment of pregnant women were chemical, psychosocial, physical-ergonomic-mechanical factors, and other work-related factors. The main adverse consequences of exposure to these factors include low birth weight, preterm birth, miscarriage, hypertension and pre-eclampsia, as well as various obstetric complications.

**Conclusion:** During pregnancy, working conditions that are considered acceptable in normal situations may not be so during this stage due to the major changes that occur during pregnancy. Many obstetric effects may have an important impact in the mother’s psychological status; therefore, it is important to optimise working conditions during this stage and to reduce or eliminate possible risks.

## Introduction

One of the most important structural changes in the labour market in recent years has been the incorporation of women into paid employment (1).

Based on the information provided by the Spanish National Institute of Statistics through the Labour Force Survey, it is estimated that the active population of Spain in 2021 reached 23 million people, of which 19 million were employed and more than 9 million were women. Disaggregated data by age and sex shows that female participation in the labour market reached 34.28% among women aged 20 to 24, and 70.05% among women aged 25 to 54. In terms of male participation, 38.49% and 80.69% for the same age groups, respectively (2).

The increase in the proportion of women in the labour force during these years has led to a parallel increase in women who continue working during pregnancy and who work for longer during pregnancy (3). In this sense, pregnancy can be considered as one more aspect of a woman’s life, one which she has to combine with the other aspects of her life, work being one of them, and where the changes that occur in women during this stage will affect her working life in one way or another. As Lee et al. (4) point out, the gestational period brings about a series of physiological, physical, hormonal, and emotional changes that facilitate adequate foetal development and that in certain cases, especially when the pregnancy is advanced, there may be certain conditions in the working environment that limit the pregnant’s ability to work. It is precisely this line that has been the focus of some studies such as the one by Larsen et al. (5) or by Katz (6), among others, whose research has aimed at analysing the risks existing in the workplace and how these can affect the normal development of pregnancy. In fact, certain working conditions such as heavy physical work, prolonged standing during the working day, carrying loads, among others, have been related to higher rates of adverse effects during pregnancy (7), as well as specific risks such as those derived from chemical products, thus requiring careful risk assessment (8).

In the light of the results obtained in the different studies, many countries, on recognising the importance of offering solutions to this issue, have proposed and adopted legislative and social measures aimed at protecting pregnant women in the workplace. In countries such as Spain, maternity protection is provided for in article 26 of Spanish Law 31/1995 of 8 November 1995 on the Prevention of Occupational Risks (9), which establishes that a risk assessment must be carried out whenever there is exposure to agents, procedures, or working conditions that could have a negative influence on the health of workers or the foetus, in any activity likely to present a specific risk.

Although current political and economic trends have favoured the adoption of safe workplaces, there are still some occupations that pose a certain risk to the normal development of pregnancy, such as the chemical industry, jobs that require long periods of standing, or continuous manual handling of loads (3, 4). Given that data show that most women continue working during pregnancy, there is a certain vulnerability to some specific work environments and conditions, where it is necessary to work on prevention in order to identify possible risk factors during this process. Therefore, the aim of this study was to evaluate the risk factors that pregnant women perceive as stressors in the work environment and the possible adverse consequences of such exposure for the normal development of the pregnancy.

## Methods

A systematic review was conducted guided by the recommendations of the PRISMA (Preferred Reporting Items for Systematic Reviews and Meta-Analyses) guidelines (10). This review was registered in PROSPERO (CRD42022344829).

The research question that marked the review was: What risk factors do working women perceive as stressors in their work environment and what are the consequences of possible exposure to them?

For an adequate development of the research question and subsequent review of the literature, the PECOT strategy was used: 1) Population: working pregnant women or women who had worked during pregnancy; 2) Effect: work-related stress; 3) Comparator: exposure to existing risk factors in the work environment; 4) Outcomes/Results: consequences of possible exposure according to the type of agent (physical-ergonomic-mechanical, chemical, psychosocial, or others: occupational-biological and environmental) and; 5) Type of studies: cohort studies, case-control studies, and quantitative studies.

This subdivision allowed selecting a combination of appropriate terms related to each of the components of the PECOT strategy so as to answer the research question posed. In this case, the descriptors used were Risk Factors, Occupational Stress, and Pregnancy, which were obtained from the MeSH (Medical Subject Headings) thesaurus. The Boolean operator used was the restricted AND combination. This was the connector that allowed the combination of the descriptors listed above to search for related literature.

The databases used were Pubmed, Web of Science (WoS), Dialnet, SciELO (Scientific Electronic Library Online), and REDIB (*Red Iberoamericana de Innovación y Conocimiento Científico*, i.e.: Ibero-American Network of Innovation and Scientific Knowledge), in which a double search was conducted by including the terms in both Spanish and English. The search in all databases was performed on 26 January 2023. In the advanced search generators of the said databases, “risk factors and occupational stress and pregnancy” was entered. The same search was performed for the Spanish terms.

The inclusion criteria for the selection of articles were: 1) articles published in English, Spanish, French, and Portuguese; 2) works with full texts accessible; 3) type of studies: cohort studies, case-control studies, and quantitative studies; 4) studies carried out in the period from 2000 to the date of the search and; 5) articles in which at least one risk factor of the physical-ergonomic-mechanical, biological, chemical, psychosocial, or environmental type could be identified. The exclusion criteria were: 1) not exceeding the scientific-technical quality criteria; 2) language reasons (language other than English, Spanish, French, and Portuguese); 3) non-pregnant working women or non-working pregnant women and; 4) non-peer-reviewed articles.

For data collection and extraction, once the keywords were agreed, two researchers searched the stated databases individually. Subsequently, they retrieved the records and entered them into the Mendeley bibliographic manager, where duplicate studies were eliminated. Then, the articles that could be included after reading the title and abstract were selected according to the previously established criteria. The selected studies were then retrieved in full text and a selection report was made individually by both researchers compiling data on authorship, country, population, type of risk factor (psychosocial, physical-ergonomic-mechanical, chemical and occupational-biological or environmental), main findings, and the result of the Joanna Briggs Institute (JBI) critical appraisal tool (11). At this point, the possibility was given to include records identified from other sources. Finally, based on the reports of the two researchers, a third author determined the studies finally included and had the power to include or not a study in case of discrepancy between the two first researchers.

The JBI critical appraisal tool for non-randomised studies was used to assess the methodological quality of the studies (11). Through this, the checklists for cohort studies (11 items), case-control studies (10 items), and quantitative studies (8 items) were used, establishing a minimum total of 8, 7, and 6 points in the different checklists, respectively.

**FIGURE 1:**
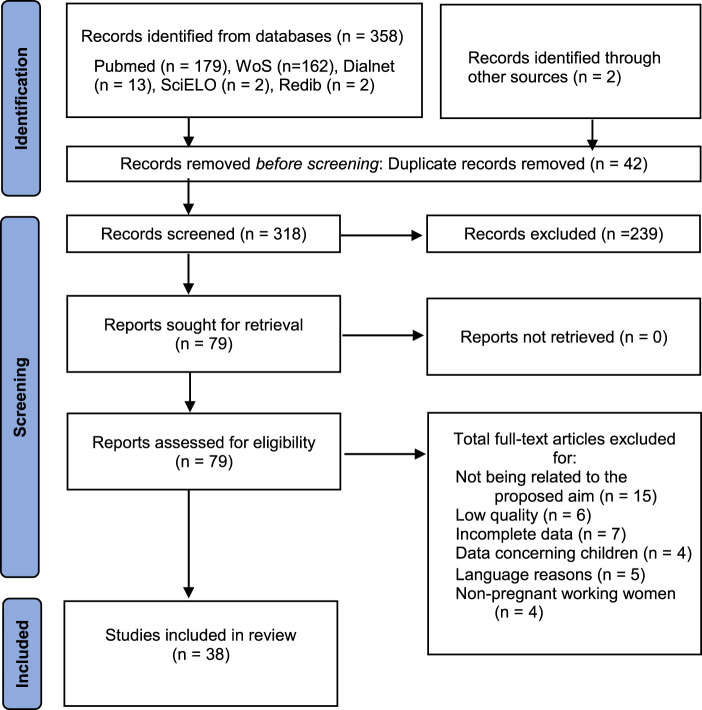
Identification of studies *via* databases (PRISMA Flow Diagram) (Occupational Risks for Pregnant Women, Spain, 2022).

The flowchart in Figure 1 depicts the sequencing of tasks and the study selection process followed in this review.

## Results

A total of 38 articles were finally selected and summarised in Table 1 (8, 12–48).

**TABLE 1:** Characteristics of the studies included in the systematic review (Occupational Risks for Pregnant Women, Spain, 2022).

Study	Country	Type of study	Population	Factors				Main findings	JBI
				PSY	ERG	CHE	OCC		
(12)	Denmark	Cohort	Women in night work (*n* = 10.047) vs*.* Women without night work (*n* = 12.697)	X			X	There is an increased risk of miscarriage after week 8 of pregnancy with an adjusted risk ratio of 1.32 (95% CI 1.07–1.62) if women had had >2 night shifts in the previous week. The adjusted risk ratio for late clinical miscarriage (week 13–22) was 1.28 (95% CI 0.70–2.34). Only 7% of miscarriages were late. The risk of miscarriage increased for each additional number of consecutive night shifts per period. Two or more night shifts in the previous week increased the risk of miscarriage after week 8 of pregnancy by 32%, compared to women who had not worked night shifts in the previous week. Thus, the number of night shifts and the number of consecutive night shifts during pregnancy weeks 3–21 showed a dose-dependent increase in risk	10/11
(13)	Denmark	Cohort	Pregnant women from the Danish National Birth Cohort (*n* = 48.890)	X				The crude prevalence of preterm birth was estimated at 4.85% in the high work stress group, 4.65% in the passive group, 5.04% in the active group, and 4.82% in the low stress group. After adjusting for covariates, there was an effect of high stress (OR 0.81, CI 0.70–0.92), compared to low-stress jobs. When studying the effect of social support, the analysis indicated a higher probability (OR: 1.39; CI: 0.86–2.23) that the child was born preterm when in the high stress group with low social support, compared to the low stress group. However, the results are not statistically significant	8/11
(14)	Cuba	Case and Control	Pregnant workers aged 19–25 (*n* = 90)	X				Pregnant workers consider their work as of high extrinsic effort and low reward, and the high demands that the work places on the pregnant woman constitute a risk factor for birth weight. In fact, a statistically significant relationship was established between perceived high demands and low birth weight, and low demands and normal weight. Lack of autonomy and of possibility to apply one’s job skills are a risk factor for these women when associated with birthweight; perceived job stress and birthweight are not. A statistically significant relationship was found between low control and low birth weight. Other statistically significant results are observed such as the association between extrinsic effort and reward, which supports the fact that, in this population group, time pressures, extra work, physically demanding work, and increasing work demands are influencing not only birth weight, but also feelings of work stress. How long the pregnant woman works during gestation in a job that generates high psychological demands, where she has low control and has to make an important extrinsic effort influences the birth weight of the newborn	8/10
(15)	Cuba	Cohort	Housewives (*n* = 429) vs*.* Women working outside the home (*n* = 521)	X			X	The difference between the frequency of low birth weight babies in housewives (18.4%) and non-housewife workers (26.9%) was almost double, although no statistically significant differences were observed between the two groups in relation to the number of gestational complications. After controlling for variables that could influence the relationship between low birth weight in each group (life situation, alcohol consumption, tobacco consumption, etc.), the effect was maintained and only a slight increase in the probability of risk (from 1.46 to 1.75) was observed in the group of non-housewife workers for having a baby weighing less than 2500 g, compared to housewives. Also, there was a decrease in the number of low birth weight births as work stress decreased (29.2% in high-stress jobs vs. 16% in low-stress jobs). In addition, high-demand, low-control work (high stress) constituted a risk for low birth weight, as did active work (high-demand, high control), with women in these types of work being 1.82 times and 2.09 times, respectively, more likely to have children below 2500 g. Passive work (low demands and low control) was not a risk factor	8/11
(16)	Portugal	Case and Control	Pregnant women with foetal delayed growth (*n* = 50) vs*.* control group (*n* = 295)	X	X			High work stress assessed showed a statistically significant association with foetal delayed growth, with the study group having a higher frequency of pregnant women with high levels of work stress than the control group (22% vs. 4%; *p* = 0.001). In addition, rotating day and night shifts showed a possible association with foetal delayed growth, although not statistically significant (*p* = 0.073). High work stress increased the risk of a pregnant woman to have a foetus weighing less than the 10th percentile by 6.65 times. Carrying or lifting weights equal to or greater than 25 kg increased the risk of a pregnant woman to have a growth-delayed foetus by 5.52 times.	8/10
(17)	Canada	Case and Control	Pre-eclampsia cases (*n* = 102)/gestational hypertension (*n* = 99) vs*.* Control (*n* = 4,381)	X	X			Consecutive work without rest days and time spent standing without walking were associated with increased risk of pre-eclampsia. Those who consecutively spent at least 1 h per day in such a position experienced a particularly higher risk. Carrying or lifting loads of at least 7 kg at least 10 times a day was associated with an increased risk of pre-eclampsia before other occupational exposures were taken into account, and pushing or pulling objects at least 5 times a day was slightly associated with pre-eclampsia and more strongly with gestational hypertension. Women who climbed stairs were twice as likely to have pre-eclampsia compared to those who never climbed stairs. Whole body vibration slightly increased the risk of pre-eclampsia, but this association was not significant. Women who never or rarely took a break and those who worked at a forced pace had a higher risk of pre-eclampsia	10/10
(18)	Denmark	Cohort	Pregnant women from the Danish National Birth Cohort (*n* = 51,874)	X	X			The risk ratios of the first sick leave episode during the 10–29 completed weeks of pregnancy were studied according to occupational exposures (working position, daily lifting 11–20 kg, daily lifting >20 kg, work shift, monthly mean of night shifts, weekly working hours). Thus, positions, daily lifts and all levels of cumulative daily lifting were associated with higher occurrence of sick leaves, compared to the reference groups. An association was also found between high work stress and increased heart rate associated to sick leaves	10/11
(19)	Netherlands	Cohort	Working women (*n* = 4865) vs. non-working women (*n* = 2696)		X			Women who worked more than 30 h/week standing, assessed during the first trimester of gestation, had an increased risk of total preterm birth. The combination of high physical workload with >32 h/week of work was not associated with total or spontaneous preterm birth, but did result in increased risk of iatrogenic total preterm birth. Women with low physical workload working <32 h/week showed a 3-fold lower risk of spontaneous or total preterm birth	10/11
(20)	China	Cohort	Women working in the petrochemical industry (*n* = 792)	X		X		Although benzene exposure was low in this population, it was significantly associated with lower birth weight. Similarly, perceived work stress was also significantly associated with lower birth weight. There is a certain interaction between benzene exposure and perceived work stress. The group with both exposures showed a 183-g reduction in birth weight compared to those with no exposure	10/11
(21)	South Africa	Quantitative	Sterilisation unit workers (*n* = 98)			X		Ethylene oxide exposure was significantly associated with an increased risk of miscarriage and of pregnancy loss. The comparison between the High Exposure vs. Low Exposure group was for miscarriage (21.1–1.3), stillbirth (13.3–3.8), and pregnancy loss (31.6–5.1)	6/8
(22)	Spain	Case and Control	Hairdressers (*n* = 89) vs*.* Control (*n* = 130)	X	X	X	X	There is no significant association between chemical exposure and risk of miscarriage. The condition reported to be most strongly associated with miscarriage was self-perceived high levels of stress (RR 2.4; 95% CI 0.2–28.3). Self-perceived work stress appears to be a more important factor for miscarriage than time spent standing and exposure to chemicals	8/10
(23)	United States	Cohort	Texas Electronic Birth Record (*n* = 385537)				X	The prevalence of hypertensive disorders during pregnancy among housewives and non-housewives was 5.03% and 6.67%, respectively. Women working in the business and management sectors and those in professions related to teaching, healthcare, legal and social services had a significantly higher risk (48%–63%) than housewives	10/11
(24)	Spain	Cross sectional	Spanish National Birth Record (*n* = 1341686)				X	The highest prevalence of preterm births is observed among mothers who are farmers (10.8%) and housewives (8.3%), and the lowest among professionals (6.6%). The highest prevalence of low birth weight is found in the service sector (3.5%), followed by industry and construction (3.4%). Female workers in the services sector have a 36% higher frequency of low birth weight at term than the rest of the sample, followed by housewives, farmers, and manual workers, with a 30% higher frequency	6/8
(25)	Mexico	Cross sectional	Hospitalised working women (*n* = 154) and Hospitalised non-working women (*n* = 154)				X	Medical complications occurring during pregnancy when comparing working and non-working women show that these occurred, in general, in equal proportion for both groups; however, there was a difference with threatened miscarriage (11.6% and 3.9%, respectively), cervicovaginitis (5.1% and 1.3%, respectively), and foetal distress (10.3% and 3.9%, respectively) for the working women group, without resulting in perinatal deaths. Work activity was not considered an important risk for prematurity and low birth weight	8/8
(26)	Taiwan	Cross sectional	3,656 pregnancies among 2,326 nurses and 111,889 pregnancies among 74,919 non-nurses				X	The rates of tocolysis (28.6% vs. 22.3%), miscarriage (6.0% vs. 5.3%), and preterm labour (8.1% vs. 4.4%) were significantly higher among nurses than among non-nurses. After adjustment for background differences, nurses had significantly higher risks for caesarean section (adjusted OR 1.12 [95% confidence interval (CI) 1.03–1.22]), tocolysis (OR 1.18 [95% CI 1.09–1.29]), and preterm labour (OR 1.46 [95% CI 1.28–1.67]) than non-nurses	8/8
(27)	Netherlands	Cohort	4,465 pregnant women	X	X	X	X	No consistent associations between any of the work-related risk factors, such as long periods of standing or walking, heavy lifting, night shifts, and working hours, nor exposure to chemicals with hypertensive disorders during pregnancy	10/11
(28)	Italy	Cross sectional	1,955 female flight attendants		X		X	The questionnaire was sent to 3,036 women with a response rate of 64% (74% for current and 48% for former flight attendants). Spontaneous abortion rates were similar for pregnancies of women in service and those who were not (12.6% vs. 11.4%; *p* = 0.58). Induced abortion rates were lower for in-service pregnancies (7.9%) compared with pregnancies of women not in service (21.11%) (*p* < 0.001)	8/8
(29)	Sweden	Cross sectional	857,010 pregnant women		X		X	Exposure to high (>85 dBA) levels of occupational noise throughout the pregnancy (full-time workers) was associated with an increased risk of the child being born small for gestational age, OR 1.44 (95% CI 1.01–2.03) compared to noise exposure <75 dBA. A similar increase was seen for low birth weight OR 1.36 (95% CI 1.03–1.80) as related to high levels of noise. No clear association was found for preterm birth. No consistent effects on birth outcome were observed in women who had worked part-time or were on leave of absence >21 days (median)	8/8
(30)	Germany	Cohort	587 employed women	X			X	Work-privacy conflict, low reward at work, and precarious working conditions significantly predicted symptoms of postpartum depression, even when controlling for lifetime depression, anxiety, education, parity, and age	11/11
(31)	Denmark	Cross sectional	343 pregnant women		X		X	The risk of preterm birth was increased in women lifting heavy loads during pregnancy (OR 1.40, 95% CI [0.88, 2.23]) but not in women with physically strenuous work (OR 0.98, 95% CI [0.66, 1.46]). The mean gestational age in the heavy-lifting group compared to the reference group was 2.4 days shorter (95% CI [0.36, 4.41])	7/8
(32)	Denmark	Cross sectional	910 pregnant women in gestational weeks 12 (baseline) and 27 (follow-up)	X			X	Work-related risk factors for sick leave were high work pace, low influence, low recognition, low job satisfaction, conflict in work-family balance, standing/walking, heavy lifting, and shift work/night shift. Health-related risk factors were burnout, stress, possibility of depression, low work ability, previous sick leave, and poor self-rated health	8/8
(8)	Egypt	Cross sectional	Working women (*n* = 730) vs. non-working women (*n* = 1,689)		X		X	There was no significant association between different work characteristics and perinatal outcomes except for that between working posture, stress, and delivery of small-for-gestational-age babies. There was an excess rate of small-for-gestational-age babies and perinatal death among the non-working group, while preterm delivery was significantly increased among those who worked throughout the whole pregnancy	8/8
(33)	Taiwan	Cross sectional	Women at 12 weeks of gestation (*n* = 172)	X	X		X	The most commonly encountered hazard was prolonged standing. The majority of women reported that the workplace provided no information on the safety or rights of pregnant women, but those exposed to at least four hazards had more access to such services (*p* < 0.05). Thirteen percent may have suffered from depressive symptomatology. Higher-level work-related burnout, lower job control, and reduced workplace support were significantly associated with possible antenatal depressive symptoms	8/8
(34)	Iran	Cross sectional	400 Iranian women	X				Employed women with pregnancies who were categorised as depression and anxiety were more likely to have low gestational age, food insecurity, several deliveries, cesarean delivery, and unintended pregnancy. Also, they were not satisfied with their infant’s sex. In addition, women with several deliveries had lower risk for postpartum depression before and after adjustment for confounders (OR = 0.92, 95% CI: 0.88–0.97, *p* < 0.001) and had lower risk for postpartum anxiety only after adjustment for confounders (OR = 0.82, 95% CI: 0.75–0.89, *p* < 0.001)	8/8
(35)	Georgia	Cross sectional	1,635 women who were employed during pregnancy	X			X	A physician or nurse had advised 27.7% (95% CI 24.5%, 30.9%) of them to stop working during pregnancy. Independent predictors of receiving this advice were hospitalisation (RR 2.3, 95% CI 1.7, 2.8) and history of previous preterm birth (RR 1.6, 95% CI 1.1, 2.2). Low birth weight (under 2500 g) occurred in 5.8% of women not advised to stop work, in 6.9% of women advised to stop work because of swelling, fatigue, stress, or another reason, and in 13.4% of women advised to stop working because of labour, high blood pressure, or vaginal bleeding (*p* < 0.001). Among women advised to stop working in the first seventh months of pregnancy, 91.7% (95% CI 88.8, 94.5) delivered at 36 or more weeks of gestation	8/8
(36)	United States	Cross sectional	2,929 women with singleton pregnancies at 22–24 weeks’ gestation		X		X	Each source of occupational fatigue was independently associated with a significantly increased risk of preterm premature rupture of membranes among nulliparous women but not among multiparous women. The risk of preterm premature rupture of membranes increased (*p* = 0.002) with an increasing number of sources of occupational fatigue-not working outside the home, 2.1%; working but not reporting fatigue, 3.7%; working with 1 source of fatigue, 3.2%; working with 2 sources of fatigue, 5.2%; working with 3 sources of fatigue, 5.1%; and working with 4 or 5 sources of fatigue, 7.4%. There was also a significant relationship (*p* = 0.01) between preterm premature rupture of membranes and an increasing number of hours worked per week among nulliparous women	8/8
(37)	Canada	Cross sectional	3,043 pregnant women	X	X		X	At 24–26 weeks of pregnancy, 31.4% (956/3,043) of pregnant women were on preventive withdrawal from work. They were more in “high-strain” (31.1% vs. 21.1%) and “Iso-strain” groups (21.0% vs. 14.2%) than those who continued to work (*p* < 0.0001). The prevalence of major depressive symptoms was higher in women on preventive withdrawal from work (10.8%; CI 95%: 8.9–12.9) compared to working women (7.1%; CI 95%: 6.1–8.3)	8/8
(38)	Canada	Case and Control	Cases (*n* = 1,242) and controls (*n* = 4,513) were selected from 43,898 women who had single livebirths	X	X		X	Results showed association of preterm delivery with demanding posture for at least 3 h per day, whole-body vibrations, high job strain combined with low or moderate social support, and a cumulative index composed of nine occupational conditions. The adjusted odds ratio increased from 1.0 to 2.0 for preterm delivery (*p* < 0.0001) and from 1.0 to 2.7 for very preterm delivery (<34 weeks; *p* = 0.0015) as the number of conditions increased from zero to four or more	10/10
(39)	Sweden	Cohort	Occupationally active mothers (*n* = 995,843)			X		Mothers who had high exposure to inorganic particles and had less than 50 days (median) of absence from work during pregnancy showed an increased risk of preterm birth (OR 1.18; 95% CI 1.07–1.30), low birth weight (OR 1.32; 95% CI 1.18–1.48), as well as low size for gestational age (OR 1.20; 95% CI 1.04–1.39). The increased risks were driven by exposure to iron particles. No increased risks were found in association with exposure to stone and concrete particles. High exposure to welding fumes was associated with an increased risk of low birth weight (OR 1.22; 95% CI 1.02–1.45) and preterm birth (OR 1.24; 95% CI 1.07–1.42)	11/11
(40)	Taiwan	Cross sectional	153 employees in their third trimester of pregnancy	X			X	Work-related feelings of stress and distress were associated with increased odds of antenatal depressive symptoms (Odds Ratio [OR] = 4.7, 95% confidence Interval [95% CI] = 1.3–19.9). Feeling tired at work (OR = 9.1, 95% CI = 2.3–47.0) and lack of support from colleagues (OR = 16.7, 95% CI = 2.9–53.1) were significantly associated with antenatal depressive symptoms	6/8
(41)	Netherlands	Cohort	4,680 pregnant women		X			There were no consistent significant associations between physically demanding work and working hours in relation to small size for gestational age, low birth weight, or preterm delivery. Women exposed to long periods of standing had lower growth rates for foetal head circumference, resulting in a reduction of approximately 1 cm (3%) of the average head circumference at birth. Compared with women working <25 h/week, women working 25–39 h/week and >40 h/week had lower growth rates for both foetal weight and head circumference, resulting in a difference of approximately 1 cm in head circumference at birth and a difference of 148–198 g in birth weight	10/11
(42)	Suriname	Cohort	384 pregnant women	X		X	X	The results showed a significant direct relationship between perceived stress and birthweight (*β* = −0.17). However, even though the relationship between perceived stress and depression was significant in all three path models (*β* = 0.61), the association between depression and birth outcomes was not significant. Perceived stress was significantly associated with community engagement (*β* = −0.12) and individual resilience (*β* = −0.12). BMI (*β* = 0.12) was also significantly directly associated with birthweight. The latent chemical construct did not show an association with the birth outcomes	10/11
(43)	United States	Cross sectional	Manicurists (*n* = 24,832), Cosmetologists (*n* = 56,373), Other working groups (*n* = 53,056), and General population (*n* = 406,025)	X	X		X	There was little evidence of increased risk for adverse birth outcomes, but an association was observed for small for gestational age among Vietnamese manicurists (OR 1.39; 95% CI 1.08–1.78) and cosmetologists (OR 1.40; 95% CI 1.08–1.83) when compared to other working women. Some maternal complications were observed, notably an increased risk for gestational diabetes (OR 1.28; 95% CI 1.10–1.50 for manicurists; OR 1.19; 95% CI 1.07–1.33 for cosmetologists) compared with the general population, which further elevated when restricted to Vietnamese workers (OR 1.59; 95% CI 1.20–2.11 for manicurists; OR 1.49; 95% CI 1.04–2.11 for cosmetologists). Additionally, an association for placenta previa was observed among manicurists (OR 1.46; 95% CI 1.08–1.97) and cosmetologists (OR 1.22; 95% CI 1.02–1.46) compared with the general population	8/8
(44)	United States	Cohort	Normotensive women (*n* = 2,422), women with preeclampsia (*n* = 44), and women with gestational hypertension (*n* = 172)	X	X		X	Women who engaged in any regular leisure-time physical activity regardless of caloric expenditure (aOR = 0.66, 95% CI: 0.35, 1.22), were unemployed (aOR = 0.64, 95% CI: 0.21, 2.00), or had non-sedentary jobs (aOR = 0.71, 95% CI: 0.37, 1.36) were at decreased risk of preeclampsia	9/11
(45)	United States	Case and control	Mothers of 511 neural tube defects cases (and 2,972 corresponding controls) and 1,163 orofacial clefts cases (and 2,969 corresponding controls)			X		The prevalence of exposure to any solvent among mothers of neural tube defects cases (*n* = 511), orofacial clefts cases (*n* = 1163), and controls (*n* = 2977) was 13.1%, 9.6% and 8.2%, respectively. Exposure to chlorinated solvents was associated with increased odds of neural tube defects (OR = 1.96, CI 1.34–2.87), in particular spina bifida (OR = 2.26, CI 1.44–3.53). No solvent class was strongly associated with orofacial clefts in these data	10/10
(46)	United States	Case and control	4,775 case and 7,734 control			X		The prevalence of occupational exposure to polycyclic aromatic hydrocarbons was 10.2% among both case and control mothers. In adjusted analysis, compared to mothers with no occupational polycyclic aromatic hydrocarbons exposure, those in the highest quartile of exposure were more likely to have offspring in the conotruncal heart defects group (OR 1.41; 95% CI 1.00–2.00) and with tetralogy of Fallot (OR 1.83; 95% CI 1.21–2.78)	10/10
(47)	Sweden	Cohort	1,080,850 pregnant women	X			X	Occupations with lower levels of decision authority were associated with increased risks of 12%–23% for hypertensive disorders of pregnancy and preeclampsia and 36%–58% for gestational diabetes compared to occupations with the highest levels of decision authority. Passive occupations had increased risks of 10% for hypertensive disorders of pregnancy and preeclampsia and 15% for gestational diabetes when compared to low strain jobs. No significant associations were found for high strain occupations	11/11
(48)	Taiwan	Cohort	10,556 working pregnant women	X			X	Among those who continued working during pregnancy, 3,850 (36.5%) mothers reported having job stress during pregnancy, and 210 (2.0%) of the children were diagnosed as having attention deficit hyperactivity disorder before 8 years of age. Compared with mothers who reported no job stress, the adjusted odds ratio of child attention deficit hyperactivity disorder was 1.91 (95% CI 1.21–3.07) for mothers with “very stressful” jobs during pregnancy and 1.53 (95% CI 1.04–2.25) for mothers with “rather stressful” jobs	10/11

PSY, psychosocial factors; ERG, physical-ergonomic-mechanical factors; CHE, chemical factors; OCC, other occupational factors; OR, odds ratio; CI, confidence interval; aOR, adjusted odds ratio.

The selected studies suggest that the main adverse consequences derived from the work activity carried out during the gestational period are low birth weight, preterm births, miscarriages, hypertension/pre-eclampsia, and gestational complications of various kinds such as miscarriage, cervicovaginitis, and foetal distress, among others (15, 25). These health problems have been related with exposure to different factors present in the workplace such as psychosocial factors, physical-ergonomic-mechanical factors, chemical factors and, lastly, other work-related factors. The main consequences of each type of exposure are detailed below.

### Consequences of Exposure to Chemical Factors During Pregnancy

In the case of the study by Ronda et al. (22) carried out in Spain, the association between the working conditions of a group of hairdressers related to exposure to chemical products and the risk of miscarriage did not reach statistical significance. In contrast, in a cohort study conducted in 2000 (20), although exposure to benzene (the chemical analysed) was low in the population studied, it was significantly associated with lower birth weight, with the group with exposure to benzene and work stress showing a reduction of 183 g. Along the same lines, the study by Gresie-Brusin et al. (21) showed that there was a significantly increased risk of miscarriage (OR = 20.8, 95% CI = 2.1–199) and pregnancy loss (OR = 8.6, 95% CI = 1.8–43.7) for pregnant women highly exposed to ethylene oxide, compared to those with low exposure. According to Norlén et al. (39), mothers who had had high exposure to inorganic particles during pregnancy showed an increased risk of preterm birth (OR 1.18; 95% CI 1.07–1.30), low birth weight (OR 1.32; 95% CI 1.18–1.48), as well as small size for the gestational age (OR 1.20; 95% CI 1.04–1.39), with increased risk related to exposure to iron particles. On the other hand, exposure to chlorinated solvents was associated with increased odds of neural tube defects (OR = 1.96, CI 1.34–2.87), in particular spina bifida (OR = 2.26, CI 1.44–3.53) (45), and working pregnant women had an increased risk of having children with conotruncal heart defects (OR 1.41; CI 95% 1.00–2.00) and with tetralogy of Fallot (OR 1.83; CI 95% 1.21–2.78) by exposure to polycyclic aromatic hydrocarbons (46).

### Consequences of Exposure to Psychosocial Factors During Pregnancy

In order to examine whether psychosocial work stress during pregnancy was related to the risk of giving birth to a preterm baby or a baby small for the gestational age, a study was conducted in Denmark by Larsen et al. (13), but the association between work stress and preterm birth was not confirmed. However, this association was indeed found in the study by Gokoel et al. (42). Likewise, Trocado et al. (16) found a possible association between low birth weight and day and night rotating shifts. Thus, high occupational stress increased 6.65 times the risk of a pregnant woman to have a foetus weighing less than the 10th percentile (16). In another study, a highly significant relationship was established between high perceived demands and low birth weight (F = 6.89, *p* = 0.001) and low demands and normal birth weight (F = 6.89, *p* = 0.001). Similarly, low control over the labour process is also a risk factor, while work stress and birth weight are not (14). In the same line, a study by Marrero, Román & Salomón found that twice as many non-housewife working women had psychosocial work stress and were 1.46 times more likely to have low birth weight babies than housewives (15). In fact, a job with high demands and low control (high stress) constituted a risk for low birth weight (OR = 1.82; 95% CI = 1.09–3.03; *p* = 0.01) as did active work (high demands and high control) (OR = 2.09; 95% CI = 1.26–3.43; *p* = 0.002) (15). In line with the findings described above, Arafa et al. (8) found a significant association between working posture, stress, and delivery of small-for-gestational-age babies, and Yeh et al. (33) found that higher-level work-related burnout, lower job control, and reduced workplace support were significantly associated with possible antenatal depressive symptoms. In some cases, work-related feelings of stress and distress were associated with increased odds of antenatal depressive symptoms (Odds Ratio [OR] = 4.7, 95% confidence Interval [95% CI] = 1.3–19.9) (40), and in the long term, it has been observed that mothers who reported having job stress during pregnancy were 1.91 times more likely (95% CI 1.21–3.07) to have a child diagnosed as having attention deficit hyperactivity disorder before 8 years of age (48).

On the other hand, Begtrup et al. (12) developed a cohort study based on a Danish national record, whose results suggest that there is an increased risk of short-term miscarriage after week 8 of pregnancy with an adjusted hazard ratio of 1.32 (95% CI 1.07–1.62) if women had had two or more nightshifts in the previous week. The authors found that for each additional number of consecutive night shifts per period, the risk of miscarriage increased. Ronda et al. (22) found that the condition with the strongest association with miscarriage was self-perceived high levels of stress (RR 2.4; 95% CI 0.2–28.3), making it the main factor for miscarriage and affecting miscarriage much more than chemical exposure.

In this sense, Pedersen et al. (32) stated that the main health-related risk factors were burnout, stress, possibility of depression, low work ability, previous sick leave, and poor self-rated health.

### Consequences of Exposure to Physical/Ergonomic/Mechanical Factors During Pregnancy

Haelterman et al. (17) conducted a study in six regions of Quebec to investigate the association between work-related physical and psychosocial factors and the risk of pre-eclampsia and gestational hypertension. In fact, standing for at least 1 h a day, carrying or lifting loads of at least 7 kg about 10 times a day, pushing or pulling objects or people at least 5 times a day, climbing stairs, and being exposed to body vibration increased the risk of pre-eclampsia (aOR) 2.5, 1.4, 2 and 1.2 times, respectively. Likewise, women with gestational pre-eclampsia had an increased risk of preterm delivery, 3 times more induction of labour, and 256 g smaller babies at birth, and 21 g and 2 times more induction of labour for those with gestational hypertension, compared to the control group. The risk of preterm birth was increased in women lifting heavy loads during pregnancy (OR 1.40, 95% CI [0.88, 2.23]), but not in women with physically strenuous work (OR 0.98, 95% CI [0.66, 1.46]), in line with what was reported in the study by Knudsen et al. (31). On the other hand, in the study by Snijder et al; (41) no consistent significant associations were found between physically demanding work and working hours with respect to low weight for gestational age, low birth weight, or preterm birth. However, it was indeed reported that women exposed to long periods of standing had lower growth rates for foetal head circumference, resulting in a reduction of approximately 1 cm (3%) of the average head circumference at birth. In the study by Nugteren et al. (27), no consistent associations were found between any of the work-related risk factors, such as long periods of standing or walking, heavy lifting, night shifts, working hours, or exposure to chemicals with respect to hypertensive disorders during pregnancy. The increasing number of hours worked per week among nulliparous women was associated with premature rupture of membranes (36). In this sense, in the study by Croteau et al. (38), an association between preterm delivery and demanding posture for at least 3 h per day, whole-body vibrations, high job strain combined with low or moderate social support, and a cumulative index composed of nine occupational conditions was found.

High levels of noise (>85 dBA) was found to be another factor possibly associated with an increased risk of the child being born small for gestational age, with OR 1.44 (95% CI 1.01–2.03), compared to noise exposure <75 dBA (29).

The results proposed by Hansen et al. (18) showed that the cumulative incidence rate of sick leaves was 36.1% from the beginning of pregnancy to 29 completed weeks of gestation. Similarly, night work hours and shift work were associated with a higher rate of sick leaves and a dose-response relationship was found between an increase in the number of monthly night shifts and sickness risks of 1.04, 95% CI 1.04–1.05. Similarly, as far as working position and daily lifting between 11 and 20 kg were concerned, there was an association with a higher frequency of sick leaves, compared to the reference groups. An association was also found between high work strain and sick leave frequency. Another study in Amsterdam concluded that prolonged standing during the first trimester was associated with an increased risk of iatrogenic preterm birth (OR = 2.9, 95% CI 1.0–4.97), but other working conditions, such as weekly working hours, physical workload, etc., were not associated with this risk (19). In contrast, the combination of high physical workload with more than 32 h of work per week resulted in three times the risk of iatrogenic preterm birth (adjusted OR = 3.42, 95% CI 1.04–8.21), compared to the reference group.

### Consequences of Exposure to Other Work-Related Factors During the Gestational Period

Ronda et al. (24) conducted a study in which they found that, in terms of preterm birth, the risk was highest among female agricultural workers, with 68% higher frequency (followed by housewives with 19% and those in the service sector with 12%). The highest prevalence of low birth weight was found in the group of women in the service sector. According to the analysed results, women working in the service sector and housewives, farmers, and manual workers have 36% and 30% higher frequency of low birth weight at term, respectively. A year later, in 2010, Rosales-Aujang found that working activity was not confirmed as a relevant risk in terms of birth weight and gestational age (25). However, there were significant differences with respect to threatened miscarriage (11.6% and 3.9%), cervicovaginitis (5.1% and 1.3%), and foetal distress (10.3% and 3.9%) for the group of working women, compared to non-working women.

There were a number of studies comparing different occupational groups. In a study, it was found that nurses had significantly higher risk of caesarean section (adjusted OR 1.12 [95% confidence interval (CI) 1.03–1.22]), tocolysis (OR 1.18 [95% CI 1.09–1.29]), and preterm labour (OR 1.46 [95% CI 1.28–1.67]) than non-nurses (26). In the study by Quach et al; (43), some maternal complications were observed, notably an increased risk of gestational diabetes (OR 1.28; 95% CI 1.10–1.50 for manicurists; OR 1.19; 95% CI 1.07–1.33 for cosmetologists), compared to the general population, which further increased when restricted to Vietnamese workers (OR 1.59; 95% CI 1.20–2.11 for manicurists; OR 1.49; 95% CI 1.04–2.11 for cosmetologists). On the other hand, in Texas, Bilhartz and Bilhartz showed that there was a strong linear relationship between occupational classification and the possible development of hypertensive disorders, and their data showed that employed women had a clear risk of about 33% higher than housewives (23).

## Discussion

Given that pregnancy implies important changes in the life of the pregnant woman that will affect, among other things, her work situation, the aim of this systematic review was to carry out a study on how work affects and what consequences it has for both the pregnant woman and the foetus. The results show that there are chemical, physical/ergonomic/mechanical, psychosocial, and other work-related factors at work that can affect the normal development of pregnancy and foetal growth.

Based on the chemical factors evaluated in the articles included in the review, exposure to inorganic particles as iron, benzene or ethylene oxide can be associated with low birth weight, miscarriage, risk of preterm birth, and even pregnancy loss (20, 39). In this regard, benzene is known to produce several toxic metabolites that affect rapidly growing cells and cause oxidative damage to cells, and also suppress cell growth, which may justify why exposure to benzene, even at very low concentrations, was significantly associated with low birth weight (49). In contrast, in the study by Ronda et al. (22), carried out in hairdressing salons, they indicated that the chemical agents analysed were not identified as risk factors for pregnant women. Thus, it may depend on the chemical agent to which the workers are exposed and/or that certain direct exposures to some specific chemicals seem to decrease in effect over the years.

In the case of occupational factors, there seems to be an association with the likelihood of hypertensive disorders, low birth weight, preterm birth, and miscarriage, among others. In this sense, belonging during pregnancy to certain occupational groups such as agricultural work, the service sector, domestic work, or manual labour in the industry and the construction sectors may have adverse effects (24). The triggering of such risks could be due, for example, in the case of agricultural workers, to the excessive physical burden of farm work, exposure to extreme temperatures, long and strenuous working hours, etc., which can increase intra-abdominal pressure and lead to uterine contractions and decreased placental blood flow. Pregnant women working outside and inside the home have also been found to be highly vulnerable in this study, as the workload and its low rewards affect them not only physically but also psychologically. According to Bilhartz and Bilhartz (23), stress can cause hypertensive disorders as well as vagal withdrawal, accompanied by decreased parasympathetic response, altered heart rate, and alterations in the maternal immune function. On the other hand, exposure to chlorinated solvents was associated with increased odds of neural tube defects (as spina bifida) and having children with conotruncal heart defects and tetralogy of Fallot (46). This could be explained by the free penetration of these substances into cell membranes, including the placenta.

In relation to physical/ergonomic/mechanical factors during pregnancy, possible negative consequences may be related to the occurrence of pre-eclampsia and hypertension, sick leaves, and preterm birth. In fact, physically demanding occupational conditions, lifting heavy loads during pregnancy and strenuous positions in early pregnancy increase the risk of pre-eclampsia, low birth weight babies, and preterm birth (17–19, 31, 50). This may be due to the limitations that physical and psychological changes produce in the woman’s body during pregnancy, such as postural changes that lead to the appearance of back pain, changes in weight that increase the feeling of fatigue and tiredness, lax ligaments that lead to a certain decrease in tolerance of loads and efforts, displacement of the centre of gravity with the consequent loss of balance, or alterations in circulation that lead to an increase in pressure in the veins, facilitating the appearance of oedemas or varicose veins, among others (17–19, 50). On the other hand, in the study by Snijder et al. (41) there were no consistent significant associations between physically demanding work and working hours in relation to small size for gestational age, low birth weight, or preterm delivery, although they attribute these results to the different types of papers analysed and to the fact that the effects of foetal growth were subtle, as these effects were not reflected at birth. Likewise, in the study by Nugteren et al. (27), no consistent associations were found between any of the work-related risk factors, such as long periods of standing or walking, heavy lifting, night shifts, working hours, or exposure to chemicals in relation to hypertensive disorders during pregnancy. These differences could be explained by the different definitions of physically demanding work that are given nowadays, which makes comparisons difficult. Only one of the studies analysed the effect of high levels of noise related to an increased risk of the child being born small for gestational age (29). Noise can cause activation of the sympathetic and endocrine systems leading to elevated blood pressure, increased heart rate, and increased levels of stress hormones. This excess cortisol over several days can cause the placenta to inhibit the conversion of cortisol into inactive cortisone, transfer unchanged cortisol to the foetus, and cause a decrease in growth (51).

The impact of psychosocial factors on the normal development of pregnancy analysed in the different articles included in the review have yielded contradictory data. Among the studies showing a significant association between psychosocial work-related stress and adverse pregnancy outcomes, the main findings are the triggering of preterm births and low birth weight babies (14, 15). Stress was also associated with working ergonomics, delivery of underweighted babies (8), antenatal depressive symptoms, and early diagnosis of hyperactivity disorder before 8 years of age (48).

This phenomenon could be explained by the fact that, when faced with a high level of stress, the body activates the hypothalamic-pituitary-adrenal axis, causing a release of corticotropin-releasing hormone, which in turn increases glucocorticoid levels and releases the stimulation of oxytocin and prostaglandins, thus favouring the onset of preterm labour (16). In other studies, Larsen et al. (13) have not found a statistically significant association and this may be due to countries with an adequate social support system and efficient legislation for the protection of pregnant women.

In line with the above, it is observed that jobs involving higher risk factors (higher exposure to physical, chemical, and psychological agents), when comparing different occupational groups, have a higher risk of caesarean section, tocolysis, preterm labour (26), and risk for gestational diabetes (43). Likewise, occupations with lower levels of decision authority were associated with increased risks of 12%–23% for hypertensive disorders of pregnancy and preeclampsia and 36%–58% for gestational diabetes, compared to occupations with the highest levels of decision authority (47). Finally, the intensity of night work may lead to an increased risk of miscarriage, especially due to sleep deprivation that causes negative neuroendocrine, immunological, vascular, and behavioural effects, as well as circadian rhythm disturbance and decreased melatonin levels (12, 52).

When work provides the opportunity to redesign jobs, to organise adjustments during pregnancy, to provide support, etc., the causal relationships found could diminish or even disappear, thus making work activity no longer an obstetric risk factor for employed women. Having knowledge of these data allows researchers to establish guidelines for future studies with the aim of clarifying the associations found. It is necessary to delve deeper through a much more complex evaluation of the subject and an evaluation that will allow institutions to determine, with these results, the intervention requirements in each case.

With regard to the limitations found in the present systematic review, it could be highlighted that, as this is a study of the different risk factors present in the working environment of pregnant women, it has not been possible to cover all the works that has been found referring to each factor. Furthermore, this study is mainly descriptive and combines data from multiple sources, which could result in an inherent reporting bias. Similarly, the review does not include conference papers, dissertations, or grey literature. Also, the measurement instruments used were different and, in some cases, the sample was small.

Based on the data obtained through this systematic review, it seems obvious that all the risk categories analysed have some negative effect on pregnancy, i.e., the presence to a greater or lesser extent of such factors can contribute to the appearance of risks that negatively affect the normal development of pregnancy.

The main adverse effects of exposure to chemical factors such as chlorinated solvents, polycyclic aromatic hydrocarbons, iron, benzene or ethylene oxide may be risk of preterm birth, low birth weight, neural tube defects, conotruncal heart defects, and miscarriage. Physical-ergonomic-mechanical factors (strenuous positions as long periods of standing, or increasing number of hours worked per week, and physically demanding conditions as whole-body vibrations, and high levels of noise) may lead to pre-eclampsia and hypertension, sick leaves, lower growth rates for foetal head circumference, premature rupture of membranes, and preterm birth. Similarly, psychosocial factors (high work demands and reduced workplace support) can lead to low birth weight, preterm birth, gestational complications, antenatal depressive symptoms, having children with attention deficit hyperactivity disorder, and miscarriage. Finally, certain occupations such as agricultural work, the service sector, nurses, etc. have an increased risk of developing hypertensive disorders, low birth weight, preterm delivery, and even foetal distress.

The results of the present review suggest that no special protection measures are being applied on a regular basis to pregnant women in the workplace, with the possibility of this being a shared responsibility between public administrations, the company, and the working women.
